# Power Benefits of High-Altitude Flapping Wing Flight at the Monarch Butterfly Scale

**DOI:** 10.3390/biomimetics8040352

**Published:** 2023-08-08

**Authors:** Chang-kwon Kang, Madhu Sridhar, Rachel Twigg, Jeremy Pohly, Taeyoung Lee, Hikaru Aono

**Affiliations:** 1 Department of Mechanical and Aerospace Engineering, University of Alabama in Huntsville, Huntsville, AL 35899, USA; mks0015@uah.edu (M.S.); rstwigg@gmail.com (R.T.); jap0025@uah.edu (J.P.); 2 Department of Mechanical and Aerospace Engineering, George Washington University, Washington, DC 20052, USA; tylee@email.gwu.edu; 3 Department of Mechanical Engineering and Robotics, Shinshu University, Ueda 386-8567, Nagano, Japan; aono@shinshu-u.ac.jp

**Keywords:** flexible wings, butterfly flight, high altitude

## Abstract

The long-range migration of monarch butterflies, extended over 4000 km, is not well understood. Monarchs experience varying density conditions during migration, ranging as high as 3000 m, where the air density is much lower than at sea level. In this study, we test the hypothesis that the aerodynamic performance of monarchs improves at reduced density conditions by considering the fluid–structure interaction of chordwise flexible wings. A well-validated, fully coupled Navier–Stokes/structural dynamics solver was used to illustrate the interplay between wing motion, aerodynamics, and structural flexibility in forward flight. The wing density and elastic modulus were measured from real monarch wings and prescribed as inputs to the aeroelastic framework. Our results show that sufficient lift is generated to offset the butterfly weight at higher altitudes, aided by the wake-capture mechanism, which is a nonlinear wing–wake interaction mechanism, commonly seen for hovering animals. The mean total power, defined as the sum of the aerodynamic and inertial power, decreased by 36% from the sea level to the condition at 3000 m. Decreasing power with altitude, while maintaining the same equilibrium lift, suggests that the butterflies generate lift more efficiently at higher altitudes.

## 1. Introduction

The phenomenon of migration, which refers to the spatial movement of species, is commonly seen among many animals, birds, and insects. Migration can occur for several reasons, such as evading predators and finding food, which can involve increased metabolic costs. Various migration strategies and migration scales can be seen in the biological world involving aerial, terrestrial, and aquatic routes.

One such insect that is known for its spectacular annual migration is the monarch butterfly, which is one of the most recognizable North American butterfly species. Its migration route extends from North America to overwintering sites in the mountains of Central Mexico, spanning up to 4000 km [1,2,3,4]. An insect with a wing span of about 10 cm traversing such an incredible distance indicates that monarchs are indeed expert flyers. However, the aerodynamic mechanisms behind the flight efficiency of their long-range migration remains poorly understood.

A key aspect that may hold the answer to understanding the monarch’s superior flight efficiency is the role played by high altitude in its migration and overwintering. Glider pilots in Canada and the United States have reported spotting monarchs at altitudes of 1200 m above ground [5]. Furthermore, the overwintering mountain ecosystems in Mexico are located at altitudes between 2900 m and 3200 m [3,4,6,7,8]. The air density at 3000 m is about 24% lower than the density at sea level [9]. Since aerodynamic forces are proportional to the air density, the drag is lower, benefiting long-range flight.

Despite the thinner air, our experimental observations of freely flying monarchs in low-density chambers, simulating higher altitudes, demonstrated that monarchs can generate enough lift to support their weight [10]. The measurements revealed that the lift coefficient generated by monarchs increased to offset the reduced dynamic pressure [10]. In particular, the flapping wing amplitude and frequency did not significantly correlate with this increase. Rather, this enhancement of the lift coefficient is strongly correlated with an increase in the effective angle of attack, measuring the wing-to-body velocity ratio.

An open question is the aerodynamic power consumption associated with the high-altitude monarch flight. Accurate power estimation from free-flight observations is difficult. To address this gap, we conducted numerical experiments of monarch flight under high-altitude conditions. The working hypothesis was that the wing flexibility and the associated fluid–structure interaction (FSI) of the wings are key to flight efficiency [11,12,13].

The objective of the current study is to model and analyze the high-altitude effects, simulated by a variation in the air density, on the aeroelastic performance of a flexible flapping wing at the monarch scale. Specifically, we varied the air density $\rho_{\mathrm{air}}$, stroke plane angle β, and the flight speed *V*. The aerodynamic lift, thrust, and power were calculated as functions of the above variables. The unsteady viscous flow physics was simulated by solving the Navier–Stokes equations.

Although the monarch wing is three-dimensional and anisotropic, we only focused on the FSI of the flexible wing in the chordwise direction. In general, the chordwise wing stiffness of insect wings is more flexible compared to the spanwise stiffness [14,15], implying that the passive pitch rotation due to the wing deformation may be essential. Furthermore, the wing pitch directly affects the aerodynamic performance through the effective angle of attack [16]. As such, we simplified the monarch wing as an elastic flat plate with a prescribed leading edge (LE) to represent the leading edge vein [14,17]. The chordwise structural parameters, i.e., Young’s modulus *E* and the wing density $\rho_{w}$, were measured from monarch butterfly wings. The aerodynamic flow field and the structural displacement field were tightly coupled through a well-validated dynamic relaxation scheme [11,12,18,19].

Body undulation and rotation play important roles in characterizing the butterfly flight [10,20,21,22,23]. Because of the large difference in the projected planform area of the wing and the body, the variations in air density are expected to have a more pronounced influence on the aerodynamic force generation of the large wings rather than the force generated from the thin body. As such, we neglected the coupling of the butterfly flight dynamics to the body undulation and rotation in this study to focus on the primary effects of the fluid–structure interaction of the wings subject to air density changes. The effects of body rotation and undulation were integrated into a single parameter of the stroke plane angle. In our model, the stroke plane angle varied by changing the angle between the freestream direction and the direction of the wing motion. The stroke plane angle could be interpreted as a parameter that integrates not only the effects of the actual stroke plane angle but also the body pitch rotation and the effective angle between the body undulation velocity and the freestream velocity in a steady approximation.

## 2. Methods

### 2.1. Experimental Measurements of the Structural Parameters of the Monarch Wing

#### 2.1.1. Wing Density

Monarch butterfly forewing volume was measured by performing a micro-CT scan of the wing. We used a MILabs U-CT micro-CT scanner at the Small Animal Imaging Shared Facility at the University of Alabama at Birmingham to scan the wing at a spatial resolution of $10\times 10^{-6}$ m. The forewing was soaked in a 1% iodine/ethanol solution for 15 h, then allowed to air dry for 20 min before being weighed and scanned. The three-dimensional forewing was reconstructed using a Hounsfield number range between −500 and −600.

#### 2.1.2. Deflection, Force, and Stiffness

**Figure 1:**
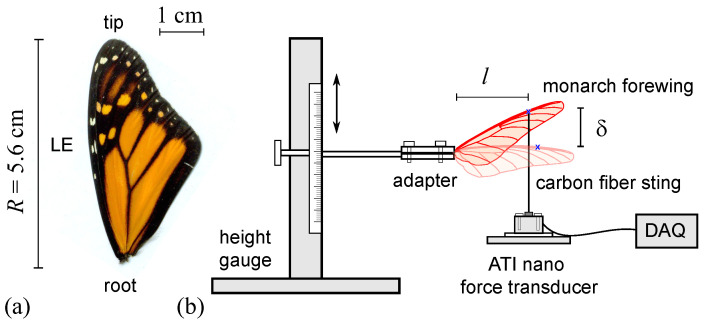
(**a**) Monarch butterfly forewing. (**b**) Butterfly wing stiffness measurement apparatus. The force balance apparatus consists of the height gauge used for measuring the deflection δ with active length *l*. For spanwise stiffness, the deflection, δ, of a wing mounted at the root was measured with a force applied at 70% of the span. For chordwise stiffness, the deflection δ of a wing mounted at the leading edge was measured with a force applied at 70% of the maximum chord.

To characterize the material properties of monarch butterfly wings, we measured the force, applied at an active length *l* per deflection δ, in both the spanwise and chordwise directions along a forewing (Figure 1a). The experimental apparatus (Figure 1b) was inspired by the pioneering study by Combes and Daniel [14]. To apply the point load to the wing, a thin and pointed carbon fiber sting was mounted to an ATI Nano 17 Titanium force transducer. The deflection was measured by attaching the wing sample to a Wixey WR200 Digital Height Gauge with a tolerance of ±0.025 mm. A 3D-printed PLA adaptor was used to connect the wing to the height gauge (Figure 1b). The measurement technique and accuracy were verified using a glass coverslip [24].

The deflection along the spanwise direction was measured with a point load to the ventral side of the wing at a location 0.7R next to the leading edge vein, where *R* is the wing length (Figure 1). Similarly, the deflection along the chordwise direction was measured using a point load at 0.7c, where *c* is the chord length. The forces were measured for deflections from 3 to 6 mm in both the spanwise and chordwise directions at a sampling rate of 200 Hz. The vertical force components were averaged once the force readings stabilized.

### 2.2. Computational Modeling of Flexible Flapping Wings at the Monarch Scale

#### 2.2.1. Wing Deformation and Motion

The monarch wings are characterized by membranes interspersed with veins (Figure 1a). The distribution of veins over the forewing is generally oriented along the spanwise direction and the veins are thicker and more rigid near the wing root; the thickness gradually reduces toward the outer edges. Modeling such large, anisotropic three-dimensional wings is a considerable challenge. That said, our force deflection measurements of monarch forewings (Section 2.1.2) suggest that the spanwise elastic modulus is about an order of magnitude higher than the chordwise counterpart. This suggests that during flight, the wings are relatively more susceptible to deforming along the chordwise direction, which acts as the wing pitch rotation, affecting the effective angle of attack [16]. As such, we focus on the wing deformations of a monarch wing model in the chordwise direction, dynamically interacting with the surrounding viscous, unsteady flow at lower-density, higher-altitude conditions.

**Figure 2:**
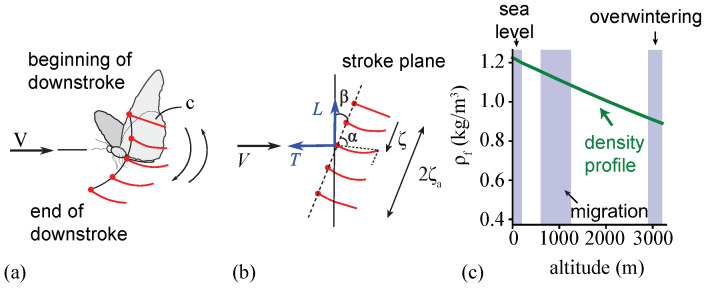
(**a**) Schematic showing flapping wing motion and (**b**) the corresponding plunge motion with an amplitude of $\zeta_{a}$. Lift and thrust directions are indicated, relative to the freestream direction. The passive pitch angle is denoted by α. Red dots indicate the LE. (**c**) Air density variation with the considered altitude. The flight altitudes at which monarchs were spotted [5] are shown in blue-shaded bands, as adapted from Sridhar et al. [26].

To simulate the wing motion, a sinusoidal motion $\zeta \left(t\right)=\zeta_{a}\cos\left(2\pi ft\right)$ with amplitude $\zeta_{a}$ and frequency *f* is imposed on the LE of the wing as a function of time *t* (Figure 2a,b). The wing deforms under the dynamic balance between the inertial, fluid dynamic, and elastic restoring forces. The resulting wing pitch is purely passive, resulting in a pitch angle of α. We do not model body undulation [22], wing–body coupling [25], or forewing and hind-wing interaction in this study.

#### 2.2.2. Governing Equations

The velocity field u and the pressure field *p* around the butterfly wings are governed by the unsteady, viscous, incompressible Navier–Stokes equations with constant density $\rho_{\mathrm{air}}$ and viscosity μ as

(1a) $$\begin{array}{cc} \nabla \cdot u & =0, \end{array}$$

(1b) $$\begin{array}{cc} \rho_{\mathrm{air}}\frac{\partial u}{\partial t}+\rho_{\mathrm{air}}\left(u\cdot \nabla \right)u & =-\nabla p+\mu \nabla^{2}u. \end{array}$$

The structural dynamics of the flexible wing along the chordwise direction *x* is modeled by the Euler–Bernoulli beam [14,17] for the wing deflection *w*, as

(2) $$\begin{array}{c} \rho_{w}h_{w}R\frac{\partial^{2}w}{\partial t^{2}}+EI\frac{\partial^{4}w}{\partial x^{4}}=pR, \end{array}$$

assuming a linear elastic wing structure with uniform thickness $h_{w}$ with *I* being the chordwise moment of inertia. Subject to the prescribed motion ζ(t) at the LE as a boundary condition, representing the leading edge vein [14,17], the fluid–structure interaction becomes two-way coupled between the fluid dynamic forces through the pressure *p*, wing inertia, and elastic bending forces.

The aerodynamic forces and moments were calculated directly by the coupled fluid (Equation (1)) and structural dynamics solver (Equation (2)), by integrating the pressure and shear forces on the wing. An in-house, structured, pressure-based finite volume solver was used in Equation (1), which governs the motion of the fluid [11,18,19,25,27,28]. Equation (2) was solved using a finite element representation of the Euler–-Bernoulli beam model. A linear Euler–-Bernoulli beam model is, in general, sufficient for the chordwise flexible airfoil [12]. The radial basis function interpolation scheme was used to deform the mesh [12,29]. The governing Equations (1) and (2) for the fluid and structure, respectively, were solved independently. The fluid–structure interaction coupling was a time-domain partitioned process. At each time step, the fluid and structural solutions were iterated until sufficient convergence was reached for the displacement of the flexible wing within an inner iteration before advancing to the next time step. The two-way coupled numerical framework has been validated against various experimental and computational data in the literature [11,12,19,25]. The details of the numerical framework, computational setup, and grid and timestep sensitivity results are further described in Sridhar et al. [26].

The wing pitch was assumed to be purely passive due to wing deformations. Although the insect flight observations suggest that insect wing pitch rotations are likely the combined outcomes of both passive and active mechanisms, there are two reasons for this assumption: (i) Butterflies, in general, are mechanically limited in their ability to actively control pitch [15], suggesting that most—if not all—of the wing pitch is due to wing flexibility; (ii) passive wing pitch results in a larger power saving than an actively actuated wing rotation, which may be more relevant to enable long-range migration. The wing planform area *S* and the chord *c* were measured for the forewings and hind wings in the overlapped configuration, as our observation of freely flying monarchs suggests that the monarchs fly with their wings in the overlapped configuration [10]. The structural parameters *E* and $\rho_{w}$, which were measured only for the forewing (Section 2.1), were assumed to be homogeneous throughout the wing.

The passive wing pitch is modeled $\alpha \left(t\right)=\tan^{-1}\left(w-\zeta \right)/c$, which is the angle between the camber line and the undeformed chord, as illustrated in Figure 2b. The passive pitch angle acts as an effective angle of attack, which is an essential aerodynamic metric. Our measurements of the wing pitch angle [23] indicate that it exhibits higher-order harmonics near the peaks. Also, the shape of the waveform is not sinusoidal. Nevertheless, to characterize the pitch angle, the passive pitch amplitude $\alpha_{a}$ and phase lag ϕ are calculated by approximating the pitch as a first-order harmonic, such that $\alpha \approx \alpha_{\mathrm{FH}}=\pi /2-\alpha_{a}\cos\left(2\pi ft+\phi \right)$.

The aerodynamic performance was evaluated using the lift *L*, thrust *T*, power consumption *P*, and propulsive efficiency η. The lift and thrust were calculated using the horizontal $F_{x}^{\prime }$ and vertical force components per unit length $F_{y}^{\prime }$ of the resultant aerodynamic force at a given stroke plane angle β (Figure 2).

The cycle averaged quantities, denoted by $\bar{\left(\cdot \right)}$, were calculated over the third motion cycle, excluding the effects of the initial transients. The lift and thrust coefficients are defined as

(3) $$\begin{array}{cc} {\bar{C}}_{L} & =\frac{\bar{L}}{\frac{1}{2}\rho_{\mathrm{air}}V^{2}c\left(2R\right)}, \end{array}$$

(4) $$\begin{array}{cc} {\bar{C}}_{T} & =\frac{\bar{T}}{\frac{1}{2}\rho_{\mathrm{air}}V^{2}c\left(2R\right)}. \end{array}$$

To measure the cost of performance, we define the power consumption as

(5) $$\begin{array}{c} P=2R\int_{0}^{c}-F_{y}^{\prime }\zeta dx+2R\int_{0}^{c}\rho_{w}h_{w}\zeta wdx. \end{array}$$

The first term models the aerodynamic power of $P_{\mathrm{aero}}$. The second term is the inertial power $P_{\mathrm{inertial}}$, associated with the inertial power related to the wing inertia and the wing motion. Finally, the propulsive efficiency η is calculated based on the cycle-averaged lift, and the power consumption as

(6) $$\begin{array}{c} \eta =\frac{\bar{L}\bar{\dot{\zeta }}}{\bar{P}}. \end{array}$$

#### 2.2.3. Design Space

To assess the effects of altitude on the aerodynamic performance at the monarch butterfly scale, the air density was varied between $\rho_{\mathrm{air}}=1.2$ kg/m^3^ and 0.91 kg/m^3^, simulating an altitude range of h=[193,3000] m.

The flight and kinematic characteristics of the monarch were based on our experimental measurements of the wing and body kinematics of freely flying monarchs in reduced density conditions [10]. The flight speed *V* and the stroke plane angle β varied between 0.5 m/s and 1.5 m/s and 0 and 60 deg, respectively. The flapping amplitude was calculated from the mean flapping amplitude $\psi_{a}$ from the experiments [10] and the range of morphological parameters from multiple butterfly specimens as $\zeta_{a}=Rr_{2}\sin\left(\psi_{a}\right)$, where $r_{2}$ is the non-dimensional radius to the second moment of wing area. A representative value was $\zeta_{a}=31$ mm and was held constant. The frequency was fixed at f=10 Hz, which was also observed in our previous experiments [10]. These experimental observations of freely flying monarchs under reduced density conditions showed that the flight speed *V* showed a statistically significant reduction with altitude, whereas the changes in the flapping amplitude and flapping frequency and their change in altitude were statistically insignificant [10]. In addition, the stroke plane angle is an important parameter that could tilt the thrust vector of flying insects.

To help analyze the results, we trained a multilayer perceptron neural network model to predict the output variables in the design space. Each design point was an FSI simulation solution. A sigmoid activation function was used for the hidden layer with 45 neurons and a linear activation function in the output layer. The network was trained with the Bayesian regularization backpropagation algorithm, which minimizes the weighted sum of square errors. Details of the employed neural network model can be found in the work by Sridhar [30].

In this study, we only focused on equilibrium–aerodynamically realizable flights, where the produced lift balances the weight of the butterfly in the appropriate Reynolds number regime. In other words, we selected and analyzed the solutions that met the following two conditions: (i) the resulting mean lift balanced the weight, i.e., $L_{\mathrm{NN}}=5\pm 0.1$ mN, based on the average butterfly weight. (ii) The resulting Reynolds number, defined as $Re=\rho_{\mathrm{air}}Vc/\mu$, matched a polynomial fit of the Reynolds number from experimental measurements.

## 3. Results

### 3.1. Wing Density and Stiffness Measurements

High-fidelity values of the wing structural parameters, i.e., the wing density and elastic modulus, in Equation (2), are essential for capturing the two-way coupled FSI of the flexible flapping wings. We experimentally measured these quantities using the methods described in Section 2.1.

**Figure 3:**
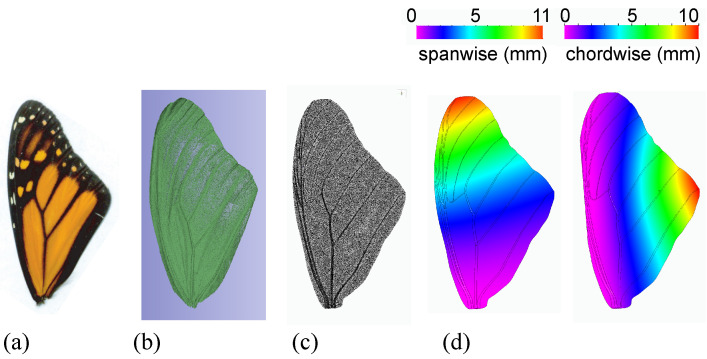
(**a**) Real monarch forewing. (**b**) Rendered image of the monarch forewing from the micro-CT scan. (**c**) Finite element model mesh of the wing. (**d**) Finite element model result of the deflection test in the spanwise and chordwise directions.

The volume of a monarch forewing was based on the reconstructed wing from the micro-CT scan (Section 2.1.1; Figure 3) shows $V_{w}=4.82\times 10^{-8}$ m^3^. The weight of the wing, measured prior to the scan, was $m_{w}=0.015$ g. The resulting wing density was $\rho_{w}=m_{w}/V_{w}=307$ kg/m^3^.

**Figure 4:**
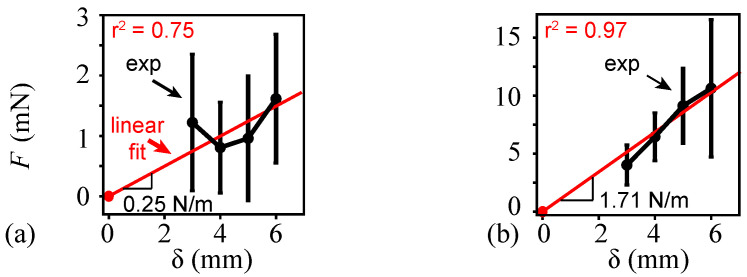
Relationship between applied force and the deflection of monarch wings in (**a**) chordwise and (**b**) spanwise directions. The mean and 95% CI from experimental measurements are shown in black. In the spanwise direction, the experimental force measurements were taken at 3,4,5,6 mm wing deflections, and the number of specimens tested were n=7,7,6,4, respectively. In the chordwise direction, the force measurements were taken at the same deflections as in spanwise directions and the number of specimens tested was n=4 at each deflection.

In the force deflection experiment (Section 2.1.2), the average force magnitudes in the spanwise and chordwise directions varied between 4.0 and 11 mN and 0.81 and 1.6 mN, respectively (Figure 4). The considered deflection range was δ=(3,6) mm. A linear fit of the experimentally measured mean forces in a chordwise direction resulted in a coefficient of determination of $r^{2}=0.75$. The mean forces required to generate the same deflection magnitudes in the spanwise direction were about six times larger. The linear fit of the measured mean forces in the spanwise direction yielded a stronger correlation with $r^{2}=0.97$.

The elastic modulus *E* was determined by creating a finite element model (FEM) and varying *E* under the applied load until the resulting deflections matched the measured deflections (Figure 3). The employed FEM assumed linear elasticity, which was justified by Combes and Daniel [14,17]. The FEM included vein structures as circular, tapered, hollow structures. More details on the FEM analysis were described by Twigg et al. [31]. The resulting average spanwise elastic modulus was E=2.5 GPa and the average chordwise elastic modulus was E=0.3 GPa.

### 3.2. Lift, Flight Speed, Thrust, and Power Variations with Altitude

**Figure 5:**
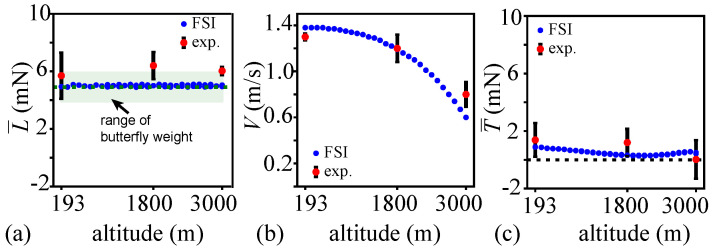
Variations of the (**a**) mean lift, (**b**) flight speed, and (**c**) mean thrust as functions of altitude for the current computation and experimental data [10]. The red dots indicate the mean values and the error bars corresponding to the 95% confidence intervals from the experimental measurements [10].

In Figure 5a, the FSI motions, resulting from the two-way coupled computational framework with chordwise flexible flapping wings at the monarch scale, had a nearly constant mean lift equal to the mean butterfly weight. These were similar to the mean lift observed in free-flight measurements [10]. The invariance of the mean lift with altitude was by design, as only equilibrium solutions were considered.

As shown in Figure 5b, the resulting flight speed decreased with the altitude. This was similar to the experimental observation of real butterflies [10]. The slight deviation of the flight speed from the current simulation results from the experimental data is likely due to the differences in the chord between the butterfly specimens used in our previous experiments [10] and the chord considered as input in the modeling. At h=3000 m, the FSI model yielded a slightly lower flight speed of V=0.6 m/s compared to the experimental mean flight speed of $V_{\exp}=0.8$ m/s.

Recall that the aim of this study was to analyze the energetics of the flights of monarch butterflies as we observed in our previous experiments [10]. As such, the numerically calculated thrust was not zero but followed the trends observed from freely flying monarchs in the experiments. The thrust (Figure 5c) was positive at all altitudes. Positive thrust implies that there is no drag and the monarch can accelerate forward. The thrust magnitude remained nearly constant; however, it was comparatively much lower than the lift. The thrust varied between T=0.3 mN and T=0.9 mN. The thrust at sea level was slightly higher than that at the h=3000 m. The low but positive thrust available at overwintering altitudes suggests that the butterflies cannot rapidly maneuver at high altitudes in the way they can at sea level. The experimental results also showed a lower thrust at the h=3000 m compared to h=193 m.

**Figure 6:**
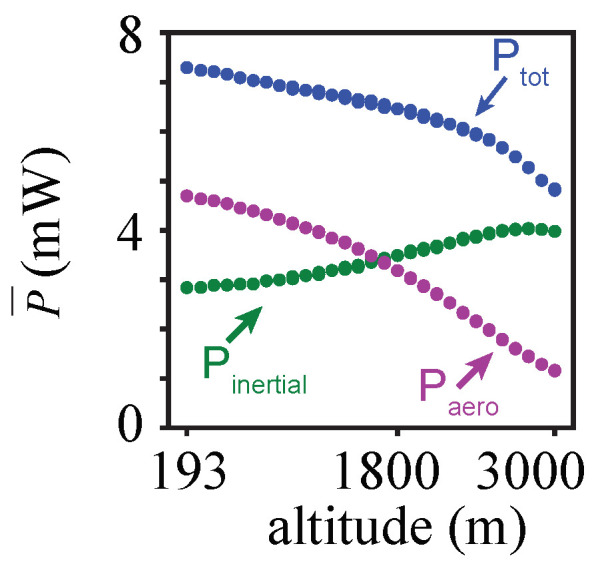
Variation of the mean power and the components obtained from the neural network as a function of altitude.

The resulting power consumption at higher altitudes (Figure 6) is much lower compared to that at sea level. For the considered equilibrium–aerodynamically realizable flights, the total power based on the FSI model decreased from P=7.3 mW at h=193 m to P=4.7 mW at h=3000 m. This power reduction is noticeable, corresponding to a decrease of 36%. The decrease in power was nearly linear with altitude.

To further analyze the trends of power consumption, the total power was split into the aerodynamic and inertial power components (Equation (5)). In general, variations in aerodynamic and inertial loads under a changing air density affect the deformation of the flexible flapping wing. In particular, the change in the air density significantly affected the aerodynamic power, which decreased between $P_{\mathrm{aero}}=4.7$ mW at h=193 m to $P_{\mathrm{aero}}=1.6$ mW at h=3000 m. On the other hand, the air density variation did not noticeably affect the inertial power, which was largely related to the wing acceleration. It remained relatively constant with a slight increase from $P_{\mathrm{inertial}}=2.9$ mW at h=193 m to $P_{\mathrm{inertial}}=4$ mW at h=3000 m. At 193 m, the contribution of aerodynamic power toward the total power is larger (64%), compared to that at h=3000 m (24%).

### 3.3. Reynolds Number, Lift and Thrust Coefficients, and Stroke Plane Angle Variations with Altitude

**Figure 7:**
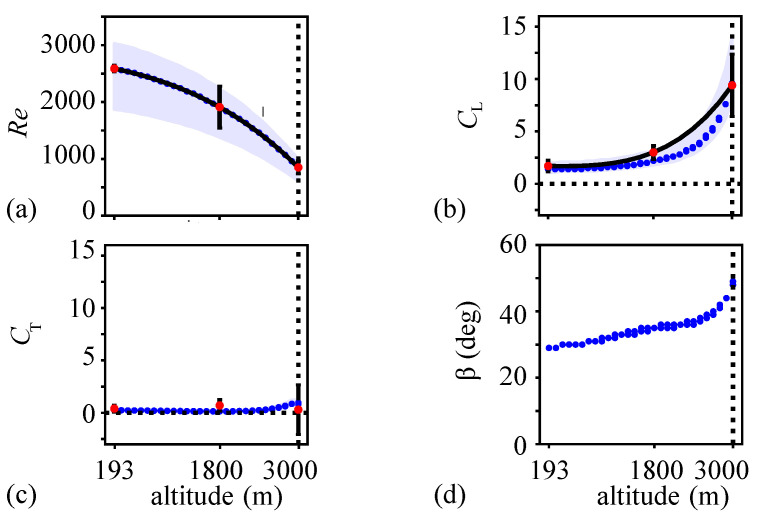
Variation of (**a**) the Reynolds number, (**b**) mean lift coefficient, (**c**) mean thrust coefficient, and (**d**) stroke plane angle. The blue dots correspond to the mean morphological parameter used in the current simulations. The shaded region in (**a**–**c**) indicates the variation due to the maximum and the minimum values in the morphological parameters in the experimental data [10]. The red dots indicate the mean values and the error bars correspond to the 95% confidence intervals from the experimental measurements [10].

The variations of the main non-dimensional quantities with altitude are illustrated in Figure 7. The shaded regions in Figure 7a–c show the ranges due to variations in the morphological parameters.

The Reynolds number reduced with altitude, i.e., Re=2588 at h=193 m to Re=850 at h=3000 m. This was a combined outcome of the decreasing air density and the flight speed (Figure 5b) with altitude. The considered FSI solutions match the experimentally observed Reynolds numbers. This was also by design, as discussed in Section 2.2.3.

The lift coefficient in Figure 7b increased slightly with altitude from $C_{L}=1.5$ at h=193 m and $C_{L}=2.3$ at h=1800 m. Beyond this altitude, the mean lift coefficient increased rapidly to $C_{L}=9.5$ at h=3000 m. The current simulation and neural network results compared well with the experimental measurements at the three altitudes. The variations due to the uncertainties in the morphological parameters remained within the uncertainties in the experiment results. Recall that the lift remained the same and equal to the weight of the butterfly. The butterfly is required to produce a much higher lift coefficient in order to fly in thin air.

The thrust coefficient in Figure 7c was nearly constant at all altitudes as expected from the thrust results (Figure 5c). The thrust coefficient remained much lower than the lift coefficient and varied between $C_{T}=0.25$ and $C_{T}=1$.

Finally, the stroke plane angle variation (Figure 7d) showed a gradual increase with altitude. The stroke plane angle at h=193 m was β=29 deg and at h=3000 m, which increased to β=49 deg. A higher stroke plane angle indicates that the butterfly flaps its wing in a plane that is oriented more closely to the horizontal.

## 4. Discussion

### 4.1. Measured Structural Parameters and Frequency Ratio

The characterization of wing flexibility in different insect species has shown that there is variety, depending on the species, due to the wing shape and venation [14,15,32,33,34]. In general, the spanwise stiffness in insect forewings is approximately one to two orders of magnitude higher than the chordwise stiffness [14], including butterfly wings [14,15,32]. This indicates an anisotropic nature to the wings present in the spanwise versus chordwise directions, as well as in the ventral versus dorsal directions [15]. The anisotropy of the wing material may allow for even greater lift generation by deforming more in one direction versus the other. The structural dynamic response of the wings could result in a reduction of overall power consumption, such that the monarchs can fly further or for longer [11].

The elastic moduli determined in this study for the monarchs exhibited a similar anisotropy. The aeroelastic implications of anisotropic monarch wings are currently unknown and requires further understanding to elucidate insect flight characteristics and promote bioinspired robot development.

The vast majority of the literature directly or indirectly cites Wainwright [35] for the insect wing density. Wainwright [35] reported a wing density value of 1200 kg/m^3^ for *Phormia* (blow flies) in 1976, cited from an unpublished source. It is unclear which method was used to measure the volume of the blowfly wings. A second source often referred to is by Jensen and Weis-Fogh [36], who also reported a density of 1200 kg/m^3^ for *Schistocerca gregaria* (flying locust), determined by cutting a wing into discrete sections, weighing each one, and measuring the area and thickness to calculate the volume.

Compared to these density measurements, the calculated density of the monarch wing, 307 kg/m^3^, is much lower. There are two explanations for the difference in the density values. One is that the previously measured 1200 kg/m^3^ was incorrect, due to less accurate measurement devices of the time, or some other error. Jensen and Weis-Fogh [36] explicitly state that their method is not very accurate. It is unclear which method was used to measure the volume of the blowfly wings. Furthermore, it is questionable if these two density measurements represent the incredibly large number of extant insect species. The second possibility is that the monarch butterfly is unique among insects, and through specialization, has developed a wing material with a low density. Alternatively, the monarch butterfly could possess modifications to the standard butterfly morphology.

The implication of the calculated wing density and elastic modulus can be discussed in terms of the frequency ratio $f/f_{1}$, where $f_{1}$ is the natural frequency in the chordwise direction. Relatively large insects are known to flap their wings far below the natural frequency [37]. Although there is no conclusive answer to the existence of optimal frequency ratio, insects exhibiting large flapping amplitudes may benefit from the fluid–structure interaction of their wings by flapping in the range $f/f_{1}=\left[1/3,0.6\right]$ [12,38], potentially near the superharmonic nonlinear resonance at $f/f_{1}=1/3$ [38].

The frequency ratio is a function of the structural properties of the wing and is independent of the fluid density. A monarch butterfly wing with an average chord length of $c=2.9\times 10^{-3}$ m, an average membrane and vein thickness near the wing root of $h_{s}=1.6\times 10^{-4}$ m, a flapping frequency of f=10 Hz, chordwise elastic modulus of E=0.3 GPa, and wing density of $\rho_{w}=307$ kg/m^3^ results in a frequency ratio of $f/f_{1}=0.33$, which is comparable to the superharmonic nonlinear resonance.

### 4.2. Wake-Capture Mechanism and Increased Efficiency in Thinner Air

**Figure 8:**
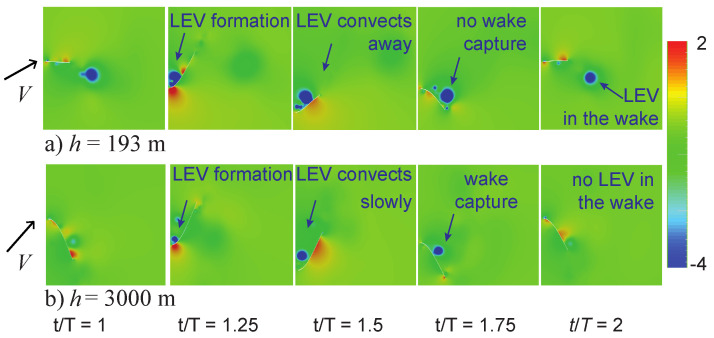
Normalized pressure $\left(p-p_{\infty }\right)/\left(\rho_{\mathrm{air}}V^{2}\right)$ contours at different time instants at (**a**) the sea level h=193 m and (**b**) the overwintering h=3000 m conditions. T=1/f is the period, such that t/T=1 corresponds to the beginning of the downstroke in the second cycle. The wing motion is vertical in this perspective.

Low Reynolds number flows are, in general, characterized by the formation, convection, and interaction with and from large coherent vortices [13,39]. Figure 8 illustrates the vortex dynamics of the flexible flapping wings at the sea level h=193 m and the overwintering h=3000 m conditions. The vortices are identified by the lower-pressure cores in the flow field [40].

At h=193 m, a leading edge vortex (LEV) forms shortly after the beginning of the downstroke. The vortex convects away from the wing due to the forward speed of the wing. As the wing flaps upwards, the LEV is already downstream of the wing. Eventually, this vortex diffuses away in the wake.

A similar LEV forms at the beginning of the downstroke for the motion at h=3000 m. However, the convection speed of the vortex is lower due to the lower flight speed. Combined with the higher stroke plane angle (Figure 7d), the LEV interacts with the wing at the end of the upstroke. This nonlinear wing–wake interaction is similar to the well-known wake-capture mechanism [13], which is utilized by smaller insects (e.g., flies and bees) in a low Reynolds number flow (typically Re<1000). The wake-capture mechanism is mainly a hover flight mechanism, where a shed vortex interacts with the wing in the returning stroke.

**Figure 9:**
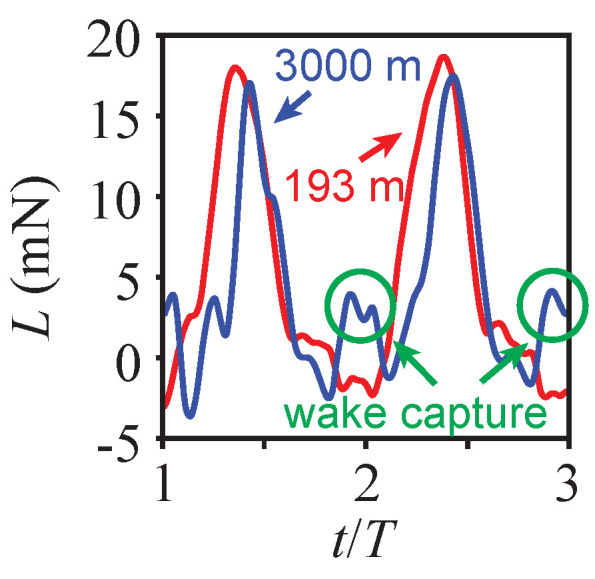
Time histories of lift as a function of t/T at the sea level h=193 m and the overwintering h=3000 m conditions.

The effect of the wing–wake interaction at the overwinter altitude of h=3000 m is shown in Figure 9. Figure 9 depicts the lift time histories at both altitudes. Recall also that the average lift is nearly the same in both motions, such that the produced lift is in balance with the butterfly weight.

During the downstroke, both motions produced a lift that was much larger than during the upstroke. The magnitude of the lift produced during the downstroke was noticeably smaller at h=3000 m. This is due to the slower flight speed, about 43% of the speed at sea level (Figure 5b), and the air density, which was 24% lower than at sea level.

On the other hand, the lift during the upstroke was higher at h=3000 m. The lift was negative during the upstroke at h=193 m, and it was positive at h=3000 m. The timing of this lift enhancement corresponded with the observed wing–wake interaction (Figure 8). These results suggest that monarchs could benefit from the wake-capture mechanism in thinner air at high altitudes. Due to the combination of the lower flight speed and higher stroke plane angle at h=3000 m, the resulting motion is akin to hovering. Furthermore, the lower air density and flight speed lower the Reynolds number to Re=850, which is within the flow regime where the wake-capture mechanisms for insects have been reported.

**Figure 10:**
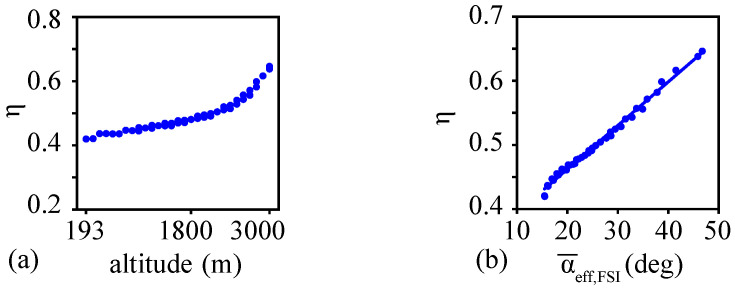
(**a**) Variation of efficiency with altitude. (**b**) Correlation between the propulsive efficiency and the mean effective angle of attack.

The resulting propulsive efficiency η (Equation (6)) increased with altitude, as shown in Figure 10a. At the overwintering altitudes, the propulsive efficiency was higher than at sea level.

To correlate the resulting efficiency with a combination of key kinematic and flight parameters, an effective angle of attack was introduced. The effective angle of attack is a key aerodynamic parameter that includes the effects of the wing motion and deformation. In this study, it was defined based on the passive pitch angle, the instantaneous wing speed, and the stroke plane angle, as

(7) $$\begin{array}{c} \alpha_{\mathrm{eff}}=\alpha -\tan^{-1}\left(h/V\right)+\beta . \end{array}$$

The mean effective angle of attack increased with altitude between 15 deg and 47 deg. The propulsive efficiency strongly correlated to the mean effective angle of attack, as shown in Figure 10b. These results show that the stroke plane angle is an essential parameter to produce sufficient lift generation and corresponding vortex dynamics in thinner air. In this model, the stroke plane angle was varied by changing the angle between the freestream direction and the flapping direction. As such, this stroke plane angle could be interpreted as a parameter that integrates not only the effects of the actual stroke plane angle but also the body pitch rotation [20,21] and the effective angle between the body undulation velocity and the freestream velocity [10] in a steady approximation.

The unsteady aerodynamics of relatively large, slowly flapping monarch wings are three-dimensional with significant wing deformations. Moreover, the undulating motion of the body is closely coupled to the wing kinematics, resulting in a very complex dynamical system. These important flight characteristics were neglected in this study. That being said, the monarch frequency ratio related to the leading edge being close to the superharmonic nonlinear resonance suggests that the combination of the fluid–structure interaction of the monarch wings (Section 4.1) and the resulting wake-capture mechanism may potentially play an essential role in enabling the long-range migration flight. The increased propulsive efficiency (Figure 10) demonstrated that using chordwise flexible wings at lower densities is consistent with this observation.

## Data Availability

The data presented in this study are available in this article.

## Abbreviations

The following abbreviations are used in this manuscript:

FEM	finite element model
FSI	fluid–structure interaction
LE	leading edge
LEV	leading edge vortex
